# The role of HHV-6 and HHV-7 infections in the development of fibromyalgia

**DOI:** 10.1007/s13365-018-0703-8

**Published:** 2019-01-07

**Authors:** Angelika Krumina, Svetlana Chapenko, Viktorija Kenina, Marija Mihailova, Inara Logina, Santa Rasa, Sandra Gintere, Ludmila Viksna, Simons Svirskis, Modra Murovska

**Affiliations:** 1 Department of Infectology and Dermatology, Rīga Stradiņš University, Dzirciema St. 16, Riga, LV-1007 Latvia; 2 August Kirchenstein Institute of Microbiology and Virology, Rīga Stradiņš University, Ratsupites 5, Riga, LV-1067 Latvia; 3 Department of Neurology, Riga Eastern Clinical University Hospital, Hipokrata 2, Riga, Latvia; 4 Department of Neurology and Neurosurgery, Rīga Stradiņš University, Dzirciema St. 16, Riga, LV-1007 Latvia; 5 Department of Family Medicine, Medical Faculty, Rīga Stradiņš University, Dzirciema St. 16, Riga, LV-1007 Latvia

**Keywords:** HHV-6, HHV-7, Fibromyalgia

## Abstract

Human herpes virus-6 (HHV-6) and human herpes virus-7 (HHV-7) are immunomodulating viruses potentially affecting the nervous system. We evaluated the influence of HHV-6 and HHV-7 infections on fibromyalgia (FM) clinical course. Forty-three FM patients and 50 control group participants were enrolled. 39.50% (*n* = 17) FM patients had light A delta and C nerve fiber damage, 27.91% (*n* = 12) had severe A delta and C nerve fiber damage. 67.44% (*n* = 29) FM patients had loss of warm sensation in feet, loss of heat pain sensation, and increased cold pain sensation (34.90%, *n* = 15 in both findings). HHV-6 and HHV-7 genomic sequences in peripheral blood DNA in 23/43 (51.00%) and 34/43 (75.50%) of samples from FM patients and in 3/50 (6.00%) and 26/50 (52.00%) of samples from the control group individuals were detected. Active HHV-6 (plasma viremia) or HHV-7 infection was revealed only in FM patients (4/23, 17.40% and 4/34, 11.80%, respectively). A statistically significant moderate positive correlation was found between A delta and C nerve fiber damage severity and HHV-6 infection (*p* < 0.01, *r* = 0.410). 23/43 patients from the FM group and control group participants HHV-6 and 34/45 HHV-7 did have infection markers. A statistically significant moderate positive correlation was found between A delta and C nerve fiber damage severity and HHV-6 infection (*p* < 0.01, *r* = 0.410). No difference was found between detection frequency of persistent HHV-6 and HHV-7 infection between FM patients and the control group. Statistically significant correlation was observed between quantitation of changes in QST thermal modalities and HHV-6 infection. There was no correlation between A delta and C nerve fiber damage and HHV-7 infection.

## Introduction

Fibromyalgia (FM) is a chronic pain disorder with unknown etiology which leads to disability and poor quality of life. The main symptoms of FM are diffuse musculoskeletal pain, fatigue, depression, sleepiness, emotional disturbance, and headache (Ballantyne et al. 2010; Lucas et al. 2006; Wolfe et al. 2010). The disorder mainly affects middle-aged females (Albin et al. 2008). The frequency of the disease increases with age, as well as symptom severity and worsening of life quality (Ballantyne et al. 2010; Balon and Wise 2015; Branco et al. 2010; Jiao et al. 2014). The prevalence of FM in the general population varies from 0.50 to 7.0% (Balon and Wise 2015).

Diagnosis is clinical and is based on 1990 American College of Rheumatology (ACR) diagnostic criteria (Hakim et al. 2010). To set up FM diagnosis, patient must have diffuse musculoskeletal pain which is presented daily for 3 months or more, in areas on both sides of the body, both above and below the waist, as well as in the spinal region, and on physical examination at least 11 of possible 18 tender points should be positive. All other possible reasons for pain have to be excluded (Hakim et al. 2010; McCarberg and Clow 2009).

In 2010, ACR presented new preliminary FM diagnostic criteria which may be easily used in primary care. The new criteria include pain and somatic symptom assessment and consider FM diagnosis, when fatigue and other somatic symptoms are more widely presented than pain. FM diagnosis is probable if the widespread pain index is more than 7 from 19 possible and symptom severity scale is equal to or more than 5 from 12 possible, or if widespread pain index is from 3 to 6 and the symptom severity scale is equal to or more than 9 (Wolfe et al. 2010).

Despite the fact that FM etiology and pathogenesis remain unclear (Albin et al. 2008), there is data that FM pain has a neuropathic nature (Dworkin and Fields 2005; Martinez-Livan 2012) and disease manifestation can follow after a specific trigger. A trigger could be a viral infection, such as hepatitis C (Kozanoglu et al. 2003; Mohammad et al. 2012; Rogal et al. 2015), parvovirus B19 (Cassisi et al. 2011), human immunodeficiency virus (HIV) (Fox and Walker-Bone 2015; Kole et al. 2013; Wiffen et al. 2013), and Epstein-Barr virus (Buchwald et al. 1987). There is no data about the possible role of human herpesvirus-6 (HHV-6) and human herpesvirus-7 (HHV-7) infection in FM development (Berneman et al. 1992). HHV-6 and HHV-7 are immunomodulating and potentially pathogenic to the nervous system and more than 90% of adults have antibodies to these viruses. HHV-6 and HHV-7 infections alter the production of chemokines and cytokines, which can significantly affect a normal immune response (Lusso 2006). After the primary infection, viruses establish a state of life-long, subclinical persistence, or latency (Yoshikawa and Asano 2000) and can be reactivated in cases of immunosuppression (Chan et al. 2001; Yao et al. 2010). Although the sites of HHV-6 and 7 during latency are not completely defined, it is known that HHV-6 can be found in peripheral blood of seropositive adults, whereas HHV-7, by T cell activation, can be reactivated from latently infected peripheral blood mononuclear cells (Kondo and Yamanishi 2007). Other human herpesviruses such as HSV-1 and HSV-2 have been described in the involvement in chronic pain, but have been connected to central nervous system involvement (Vartiainen et al. 2009).

Studies which showed a reduction of dermal unmyelinated nerve fiber bundles in skin samples of patients with FM, comparing with the control groups, support the assumption that FM pain has a neuropathic nature (Uceyler et al. 2013).

Quantitative sensory testing (QST) methods are used to evaluate the dysfunction of small fibers (Cruccu et al. 2010). Thermal and mechanical incentives are used to measure different sensor thresholds that correspond to different receptors, peripheral nerve fiber tips, and different CNS tracts (Hansson et al. 2007). Abnormal test results indicate sensory tract dysfunction from receptor to sensory cortex (Shy et al. 2003). Based on studies, QST can be used in cases of diabetic neuropathy, small fiber neuropathy, toxic neuropathy, uremic neuropathy, and other neuropathic pain syndromes (Shy et al. 2003), and probably to predict a response to treatment (Gustorff et al. 2013). In all cases, QST results should be interpreted together with a clinical pattern and another diagnostic test (EMG, nerve or skin biopsy, CT, MRI, etc.), because according to QST dates, it is not possible to determine the level of dysfunction (Shy et al. 2003). The German Research Network on Neuropathic pain has developed a standardized QST protocol which includes 13 parameters for thermal, mechanical stimulus detection, and pain perception (Rolke et al. 2006) and which analyses the functioning of A beta, A delta, and C nerve fibers (Sydney and Conti 2011).

The assumption that FM pain has a neuropathic nature increases the value of QST as an additional diagnostic tool.

Previous studies have shown the difference of thermal QST results in FM patients, comparing with a control group, but the results are heterogeneous (Blumenstiel et al. 2011; da Silva et al. 2013; Klauenberg et al. 2008; Pavlakovic and Petzke 2010; Plauf et al. 2009; Tampina et al. 2012; Uceyler et al. 2013).

## Methods

### Population

The research group included 43 FM patients undergoing outpatient treatment. The FM diagnosis was established based on ACR 1990 and 2010 diagnostic criteria (Hakim et al. 2010; McCarberg and Clow 2009; Wolfe et al. 2010).

Clinical signs were excluded from patients with previously known polyneuropathy and other neurological diseases, including lumbar and cervical radiculopathy. Patients did not have type 1 or type 2 diabetes mellitus, toxic anamnesis (e.g., alcohol or drug overuse), psychiatric disease, and acute or chronic CMV, HCV, and HIV infection.

From 43 patients included in the research, 42 were female and 1 was male with the average age 51.80 (min. 24 years, max. 72 years, SD ± 10.19) and mean duration of diffuse pain—9.30 years (min. 1.5 months, max. 35 years, SD ± 10.20).

Research was reviewed and approved by Riga Stradins University Ethics Committee.

### Assessment of clinical symptoms

Patients were interviewed with several questionnaires to evaluate the severity of their pain, their level of fatigue, and the presence of other somatic symptoms, as well as the disorder influence on the daily functioning and quality of their lives.

Pain severity was estimated using 2010 ACR diagnostic criteria widespread pain index with a maximum score of 19. Symptom severity score apprises fatigue level, waking unrefreshed, cognitive symptoms, and the presence of other possible somatic symptoms, with maximal score 12 in total (Wolfe et al. 2010).

In addition, chronic fatigue was estimated by the Fatigue Severity Scale (FSS) with a maximum score of 63. Results equal to or more than 36 indicate a presence of chronic fatigue (Valko et al. 2008).

The influence of pain and other symptoms on daily functioning was measured using the Fibromyalgia Impact Questionnaire (FIQ) with a maximum score of 100. Based on studies, a score of around 50 corresponded to the average rating for a patient with FM; a score above 70 indicated a severe course of the disease (Assumpacao et al. 2010; Bennett 2005).

### Quantitative sensory testing

From 13 QST parameters which were presented by the German Research Network (Rolke et al. 2006), we used four thermal detection thresholds for the perception of cold and warm and thermal pain thresholds for cold and hot stimuli. These parameters analyzed thinly myelinated A delta fibers, and non-myelinated C fibers, which are responsible for pain transmission and spinothalamic pathway (Rolke et al. 2006; Sydney and Conti 2011). Sensory disturbances were examined in 43 patients using a *PATHWAY* device (Medoc Ltd.). This device generates a defined and calibrated impulse within certain limits (Cruccu et al. 2010). During the test, a probe was attached to the skin of the patient that warmed or cooled it. From the adapting temperature (32 °C), the probe started to generate certain caliber thermal stimulus—temperature continuously rises or falls. The patient stops the stimulus rising (or falling) when a predefined sensation (temperature perception or temperature induced pain) is reached. The temperature during the test can rise up to + 50 °C and fall down to + 20 °C. If the maximum or minimum temperature limit is reached, the device stops and returns to adapting the temperature. During the test, patient cannot see the screen (Rolke et al. 2006).

Patients were tested on hand and foot dorsal surfaces: warm and cold perception thresholds were measured four times each in course, cold and heat pain perception thresholds were measured three times each in course.

Based on studies, hands were more sensitive than feet for cold and warm detection thresholds. In healthy subjects, mean warm detection threshold varied from 32.6 to 33.4 °C on the hand, from 33.7 to 36.4 °C on the foot, and the cold detection threshold was around 31 °C on the hand and from 29.3 to 31.4 °C on the foot. Studies did not show a significant difference between foot and hand for heat-induced and cold-induced pain thresholds. The mean heat-induced pain threshold in healthy subjects was around 43 °C and cold-induced pain threshold varies from 11.7 to 16.2 °C (Dyck et al. 1993; Hagander et al. 2000; Hansen et al. 1996). Cold-induced pain is the most variable modality which is the most difficult to evaluate.

To compare a single patient’s QST data with group, mean of age and gender matched healthy controls (HC), patients’ data were *Z*-transformed for each single parameter by using the following expression: *Z*-score = (Mean_single patient_ − Mean_controls_) / SD_controls_.

Z-score value higher than 1.95 and lower than − 1.95 indicates significant changes in thresholds comparing with the control group. Z-score of zero represents a value corresponding to the group mean of the HC subjects (Plauf et al. 2009).

### Detection of HHV-6 and HHV-7 infection markers

The presence of persistent HHV-6 and/or HHV-7 infection markers and infection activity phase was examined in all 43 patients with FM and 50 control group individuals.

Nested polymerase chain reaction (nPCR) was used to detect viral genomic sequences in DNA isolated from a whole peripheral blood (WPB) (marker for persistent latent infection) and cell-free blood plasma (marker of active infection). The total DNA was isolated from WPB using the standard phenol-chloroform extraction. The QIA amp DNA Blood Mini Kit was used to extract DNA from plasma. To ensure the quality of the DNA, a β-globin PCR was performed. nPCR amplification to detect the presence of virus genomic sequences was carried out in the presence of 1 μg of WPB DNA and 10 μl of plasma DNA (corresponding to 100 μl of plasma). HHV-6 and HHV-7 DNA were detected in accordance with Secchiero et al. 1995 and Berneman, Z.N., respectively. Positive controls (HHV-6 and HHV-7 genomic DNA; ABI, Columbia, MD, USA) and negative controls (DNA obtained from practically healthy HHV-6 and HHV-7 negative donors and reaction without template DNA), as well as water controls, were included in each experiment. In our experiments, the sensitivity of HHV-6-specific primers corresponded to three copies of the HHV-6 genome and the sensitivity of HHV-7-specific primers to one copy of the HHV-7 per reaction (Tomsone et al. 2001; Kozireva et al. 2009).

## Quantitative real-time polymerase chain reaction

HHV-6 and HHV-7 loads were detected in all WPB DNA samples positive for viral genomic sequences of the patients with FM and control individuals using HHV-6 Real-TM Quant kit (Sasace, Biotechnologies, Italy) and Realquality RQ-HHV-7 kit (AB ANALITICA, Italy), and BioRad CFX96 Real-time PCR System (BioRad, USA) according to the manufacturers’ recommendations. The estimate of the median viral loads was carried out by taking into account the nPCR results.

## mRNA expression detection

RNA was isolated from peripheral blood mononuclear cells (PBMC) (that was positive for HHV-6 and/or HHV-7 DNA) of the patients with FM and control individuals using TRI Reagent (Sigma-Aldrich), and cDNA synthesized using innuSCRIPT One Step RT-PCR SyGreen Kit (Analytik Jena, Germany), according to the manufacturers’ recommendations. Acquired cDNA samples were used in PCR to detect HHV-6 U89/90 mRNA (regulatory immediate-early or α gene) and HHV-7 U57 mRNAs (coding the major capsid protein) expression.

## Statistical analysis

All the graphs, calculations, and statistical analyses were performed using GraphPad Prism software version 8.0 for Mac (GraphPad Software, San Diego, CA, USA). To test whether the collected numerical data are normally distributed, the D’Agostino and Pearson, Anderson-Darling, and Shapiro-Wilk normality tests were applied. *Z*-transformation of the QST data was performed using the following expression  –$Z=\left(x=u\right)\kern-.3pc$/σ 푧=(푥−휇)/휎, where $x$ 푥 corresponds to the individual variable from the datasets that must be normalized with *z*-transformation, while μ 휇 and σ 휎 are the control group mean and standard deviation (SD), respectively. *Z*-value equal or higher than 2, also equal or lower than – 2, indicates that the variable significantly differs from the control group mean. The comparison of means between different groups of numerical variables was performed using one-way ANOVA. Homogeneity of variances was tested using Brown-Forsythe and Bartlett’s tests, and in a case of unequal SDs, Brown-Forsythe and Welch ANOVA tests were applied. If data were not normally distributed, the comparison of medians between different groups was switched to non-parametric one-way ANOVA on ranks or Kruskal-Wallis test followed by two-stage step-up method of Benjamini, Krieger, and Yekutieli as post-hoc test. Because most of the data was distributed not normally, results are expressed as median and interquartile range (IQR) as dispersion characteristic, and *p* value less than 0.05 (*p* < 0.05) was considered as statistically significant.

## Results

### FM symptom presentation

More frequent areas of pain, assessed by the 2010 ACR FM diagnostic criteria, were lower and upper parts of the back (93% of 43) and shoulders (93% of 43). More than 80% of 43 patients reported pain in different parts of their legs, with more than 70% in arms. Mean WPI was 12.30 from 19 possible (min. 3, max. 18, SD ± 3.60; *p* < 0.0001).

Assessed by the 2010 ACR FM diagnostic criteria symptom severity score, more than 80% of all patients reported thinking or remembering problems, muscle weakness, headache, numbness, dizziness, insomnia, or nervousness. Fifty to 70% of all patients had frequent urination, hearing difficulties, shortness of breath, pain/cramps in the abdomen, depression, nausea, chest pain, blurred vision, fever, dry mouth, as well as ringing in ears (dates of the frequency of somatic symptoms are available in Table 1).

**Table 1:** Somatic symptom frequency assessed by 2010 ACR diagnostic criteria (number of patients, percentage of all examined patients)

Dry mouth	32 (71.1%)	Frequent urination	26 (57.8%)
Pain in upper abdomen	21 (46.7%)	Hives/welts	11 (24.4%)
Diarrhea	16 (35.6%)	Ringing in ears	33 (73.3%)
Muscle weakness	39 (86.7%)	Bladder spasms	11 (24.4%)
Headache	38 (84.4%)	Muscle spasms	31 (68.9%)
Hearing difficulties	24 (53.3%)	Dry eyes	19 (42.2%)
Raynauld’s	11 (24.4%)	Pain/cramps in abdomen	23 (51.1%)
Depression	34 (75.6%)	Loss of appetite	17 (37.8%)
Constipation	15 (33.3%)	Rash	11 (24.4%)
Dizziness	37 (82.2%)	Vomiting	6 (13.3%)
Sun sensitivity	22 (48.9%)	Hair loss	20 (44.4%)
Nausea	25 (55.6%)	Heartburn	23 (51.1%)
Nervousness	39 (86.7%)	Fever	29 (64.4%)
Chest pain	23 (51.1%)	Numbness/tingling	39 (86.7%)
Remembering problems	40 (88.9%)	Blurred vision	31 (68.9%)
Itching	21(46.7%)	Oral ulcers	5 (11.1%)
Insomnia	38 (84.4%)	Loss/change in taste	11 (24.4%)
Shortness of breath	29 (64.4%)	Irritable bowel syndrome	17 (37.8%)
Wheezing	14 (31.1%)		

Accessed by the symptom severity score, all patients reported fatigue. Twenty-two (51.20%) out of 43 patients assessed their fatigue as being severe, pervasive, continuous, and life disturbing; 20 (46.50%) patients described their fatigue as being moderate, often presented and/or at a moderate level, and only 1 (2.30%) patient described fatigue as being slight. Mean symptom severity score was 8.10 out of 12 (min. 5, max. 12, SD ± 1.50; *p* < 0.0001).

Fatigue level was additionally evaluated using the FSS, 41 (95.30%) out of all patients had chronic fatigue, score more than 36 (*p* < 0.0001); and 24 (8.7%) patients had the maximum score of 63. The mean score was 53.90 (min. 25, max. 63, SD ± 8.80; *p* < 0.0001).

Quality of life was assessed by FIQ. Seven (16.30%) patients scored quality of life less than 50, 22 (51.20%) out of 43 patients between 50 and 70, and 14 (32.5%) more than 70, which indicates a severe course of the disease (*p* < 0.0001) (Assumpacao et al. 2010; Bennett 2005). The mean score was 64.7 out of 100 in total (min. 26, max. 95 SD ± 14.30); (*p* < 0.0001).

## Quantitative sensory testing

Quantitative sensory testing was performed on 43 patients. We analyzed the average thermal QST results in feet and arms separately because most small fiber neuropathies had occurred in a length-dependent fashion and primarily affected lower extremities (Hovaguimian and Gibbons 2011; Themistocleus et al. 2014).

Eleven (25.60%) out of 43 patients had significant hypoesthesia (reduced or partial loss of sensory function, which means both cold and warm perception thresholds may be increased) in feet, of which two patients also had significant hypoesthesia in hands (cold perception threshold less than 27 °C). In 20 (46.50%) out of 43 patients, significant hypoesthesia in feet was detected, in one (2.30%) patient, there was significant hypoesthesia in hands (warm perception thresholds more than 40 °C). None of the patients had hypoesthesia in both their feet and hands.

In 28 (65.10%) out of 43 patients, significant hypoalgesia in feet was detected, and in 24 (55.80%) out of 43 patients, there was significant hypoalgesia in hands (heat pain threshold more than 45 °C). In 21 (48.80%) out of 43 patients, heat hypoalgesia in both hands and feet was detected, and only in 3 (7%) out of 43 patients, heat hypoalgesia was only in hands.

**Fig. 1:**
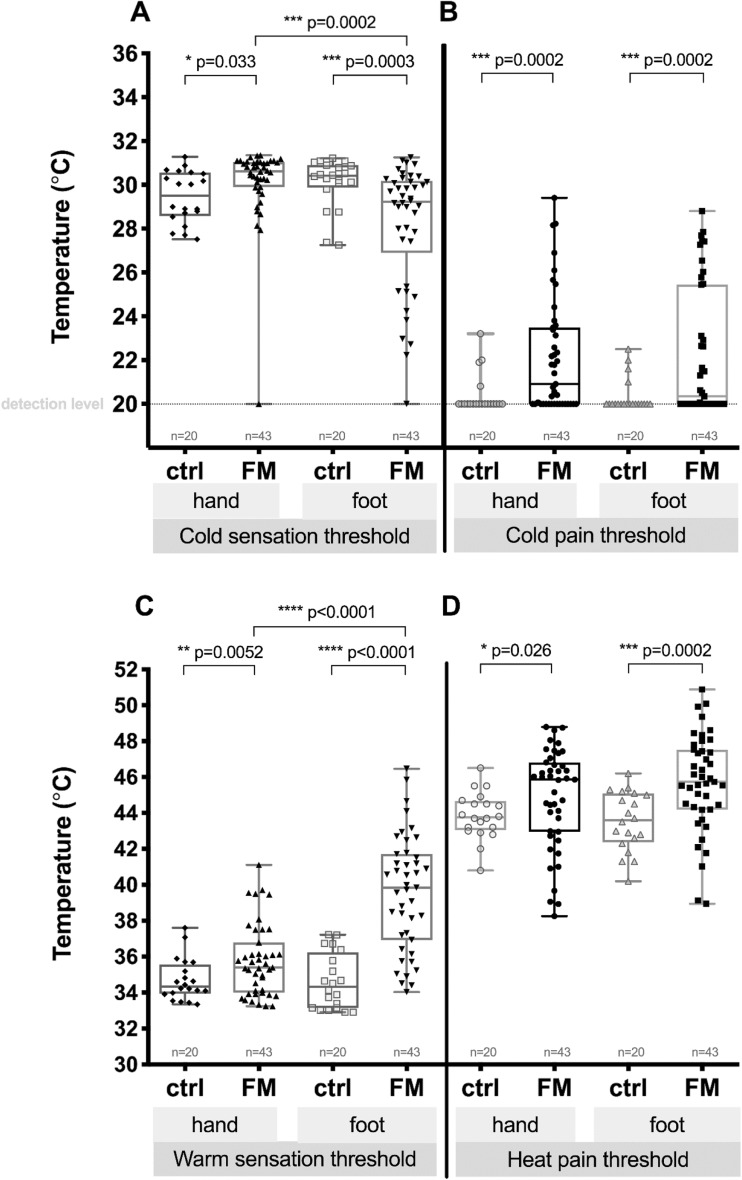
Cold sensation (**a**), cold pain (**b**), warm sensation (**c**), and heat pain (**d**) thresholds on hands and feet in control subjects (ctrl) and FM patients. Results are displayed as combined box plot (median and interquartile range (IQR) as dispersion with Min to Max bar) and scatter graph of mean values from the measurements on both hands or feet. Fine dotted line shows detection level

In 4 (9.30%) out of 43 patients, significant hyperalgesia in hands, and in 2 (4.70%) of them, significant hyperalgesia in feet was observed (heat pain threshold less than 40 °C). Eventually, in 35 (81.40%) out of 43 patients, there was significantly modified heat pain threshold in hands, in feet, or in all tested surfaces. In 5 (11.60%) out of 43 patients, significant cold hyperalgesia in feet was detected, and 3 (7%) out the 43 patients had also significant cold hyperalgesia in hands (cold perception threshold more than 27 °C). In 22 (51.20%) out of 43 patients, a cold pain threshold of less than 20 °C was observed in hands, and in 23 (53.50%) out of 43, in feet (QST graphical representation available in Fig. 1).

In 4 (9%) out of 43 patients, no significant changes in any thermal QST modality were observed; in 17 (39.50%) out of 43, significant changes in one thermal QST modality were detected (in hands, in feet, or in both hands and feet), which indicates on light A delta and C nerve fiber damage. In 10 (23.30%) out of 43 patients, there were significant changes in two thermal QST modalities detected, which indicate on moderate A delta and C nerve fiber damage. In 12 (27.90%) out of 43 patients, significant changes in three thermal QST modalities were observed. None of the patients had significant changes in all four thermal QST modalities. Changes in three and more thermal QST modalities indicate on severe A delta and C nerve fiber damage.

**Fig. 2:**
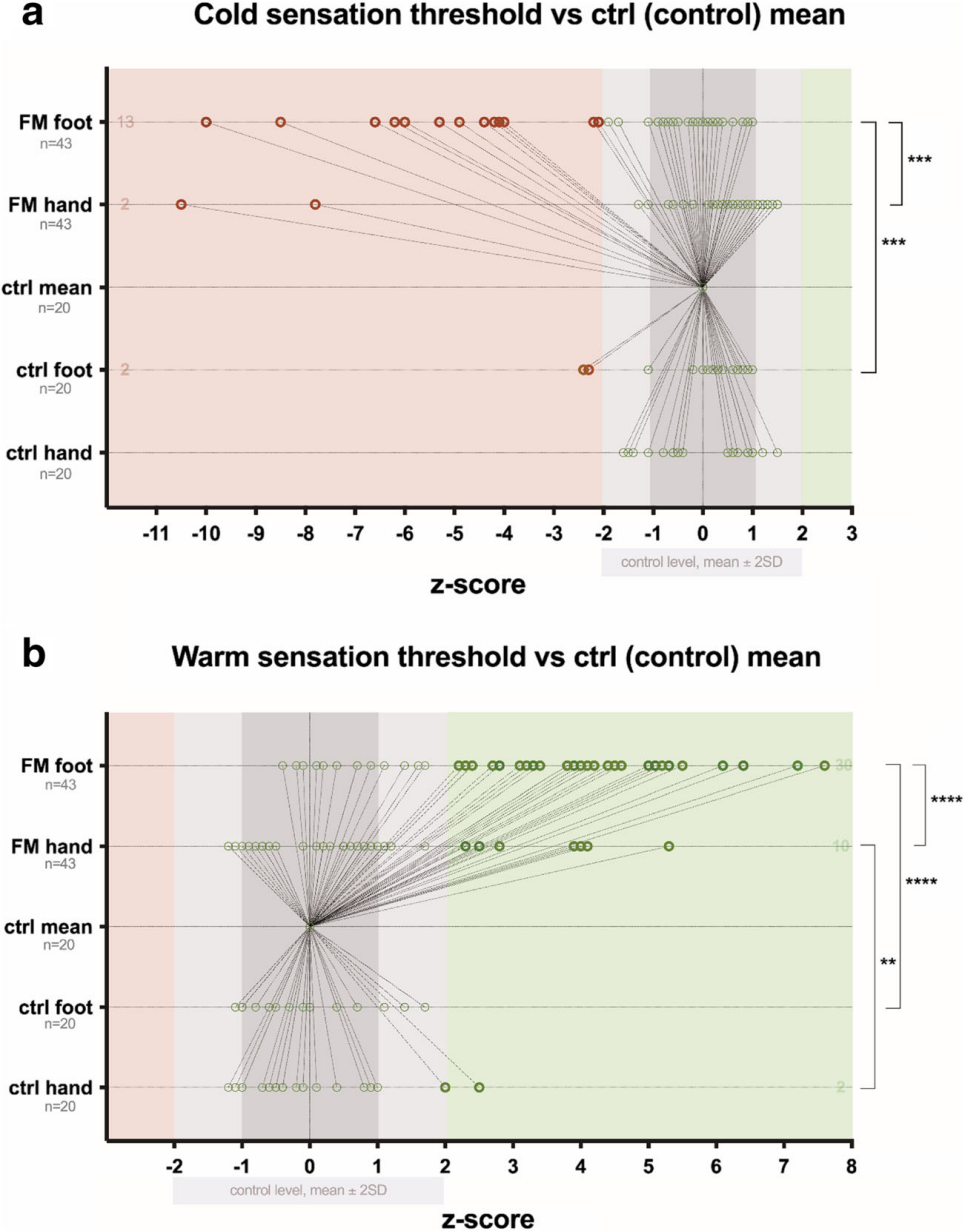
Distribution of *z*-scores vs control level in healthy subjects (control) and FM patients. **a** Cold sensation threshold. **b** Warm sensation threshold. *Z*-values that significantly differ are marked as bold shape

**Fig. 3:**
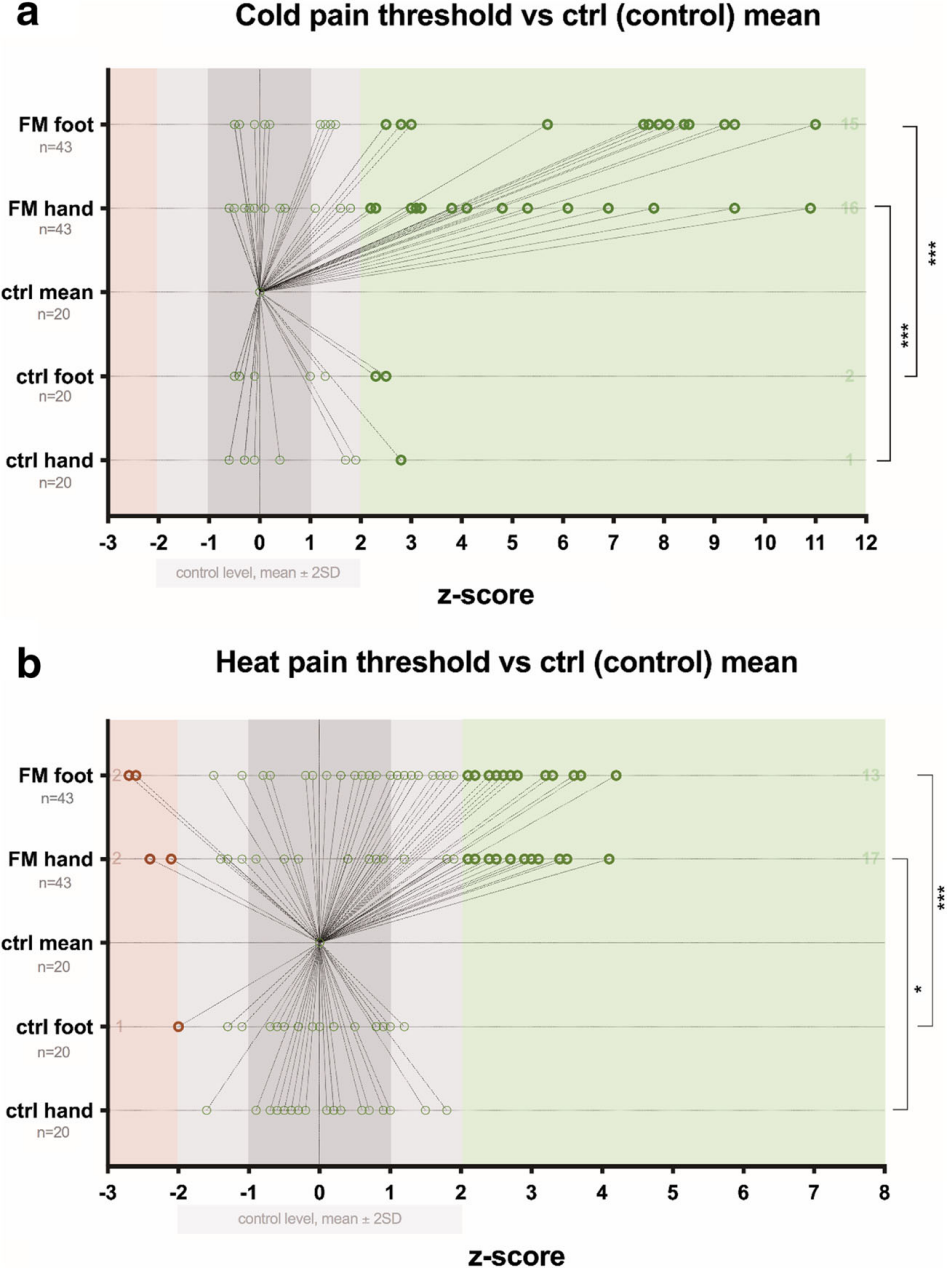
Distribution of *z*-scores vs control level in healthy subjects (control) and FM patients. **a** Cold pain threshold. **b** Heat pain threshold. *Z*-values that significantly differ are marked as bold shape

By analyzing *z*-score mean values, 29 (67.40%) out of 43 patients had loss of warm sensation in feet and 10 (23.30%) out of 43 in hands, comparing with healthy controls. Fifteen (34.90%) out of all patients had loss of heat pain sensation in feet, and 13 (30.20%) loss of heat pain sensation in hands, comparing with healthy controls. Two (4.70%) out of patients had increased heat pain sensation in feet and 6 (14%) increased pain sensation in arms, comparing with healthy controls. In total, in comparison with the control group, 17 (39.50%) out of 43 patients had significant changes in heat pain threshold in feet, and 19 (44.20%) significant changes in heat pain threshold in hands. Thirteen (30.20%) patients had loss of cold sensation in feet, and 2 (4.70%) loss of cold sensation in hands, comparing with healthy control. Fifteen (34.90%) out of 43 patients had increased cold pain sensation in feet, and 13 (30.20%) increased cold pain sensation in hands (a graphical representation of thermal QST threshold changes in FM patients comparing with healthy control is available in Figs. 2 and 3).

## Detection of HHV-6 and HHV-7 infection markers

Qualitative nPCR testing was performed on 43 patients. Roseoloviruse genomic sequences were detected in 36 out of 43 (83.70%) FM patients DNA WPB samples versus 10 out of 20 (50%) control group individuals (*p* = 0.013). No difference in frequency of single HHV-6 and single HHV-7 infection was detected between FM patients (3/43, 7% and 19/43, 44.20%, respectively) and control group individuals (1/20, 5% and 8/20, 40%, respectively). However, a significantly higher rate of concurrent (HHV-6 and HHV-7) infection was diagnosed in FM patients in comparison with control group individuals (14/43, 32.60% versus 1/20, 5% respectively; *p* = 0.024).

Plasma viremia (a marker of active viral infection) was revealed only in patients with FM (6/36, 16.70%). From them, 2/3 had single HHV-6, 2/19 single HHV-7, and 2/14 concurrent infection. In patients with concurrent infection, both HHV-6 and HHV-7 DNA in plasma were detected.

Median HHV-6 and HHV-7 loads were higher in WPB DNA samples in FM patients (815.8 [ranges from 1126.5 to 366.24] and 311 [ranges from 435.8 to 63.3] copies/10^6^ cells compared to the control group individuals (125.5 [range 192.5–52.5] and 173.12 [range 281.37–66.73] copies/10^6^ cells, respectively). HHV-6 genomic sequences were detected in 23 out of 43 (51.10%) FM patients versus 3 out of 5 (6%) control group individuals (*p* < 0.001). HHV-7 infection in FM patients and control group individuals was 34 out of 43 (75.50%) and 26 out of 50 (52%); *p* = 0.020 respectively. HHV-6 plasma viremia (a marker of active viral infection) was revealed only in patients with FM (4/23, 17.40%) and HHV-7 in (4/34, 11.70%) patients. Median HHV-6 and HHV-7 load levels were higher in FM patients [range 6104.2–67.16] and 583 [range 953–< 10] copies/10^6^ cells, respectively, compared to the healthy individuals (125.5 [range 192.5–52.5] and 181.52 [range 281.37–86.63] copies/10^6^ cells, respectively).

## Relation between QST and the presence of viral infection markers

To estimate correlation, QST results and the presence of viral infection markers results were transformed. For QST, 0 indicated no changes in any modality, 1 - abnormal QST results in one modality, 2 - abnormal results in two modalities, and 3 - abnormal results in three or more modalities. For the presence of viral infection markers, 0 indicated that no viral genomic sequences are presented, 1 - presence of latent virus infection markers and 2 - presence of active virus infection markers. Distribution of modalities with respect to FM and to HHV infection can be seen in Fig. 8.

**Fig. 4:**
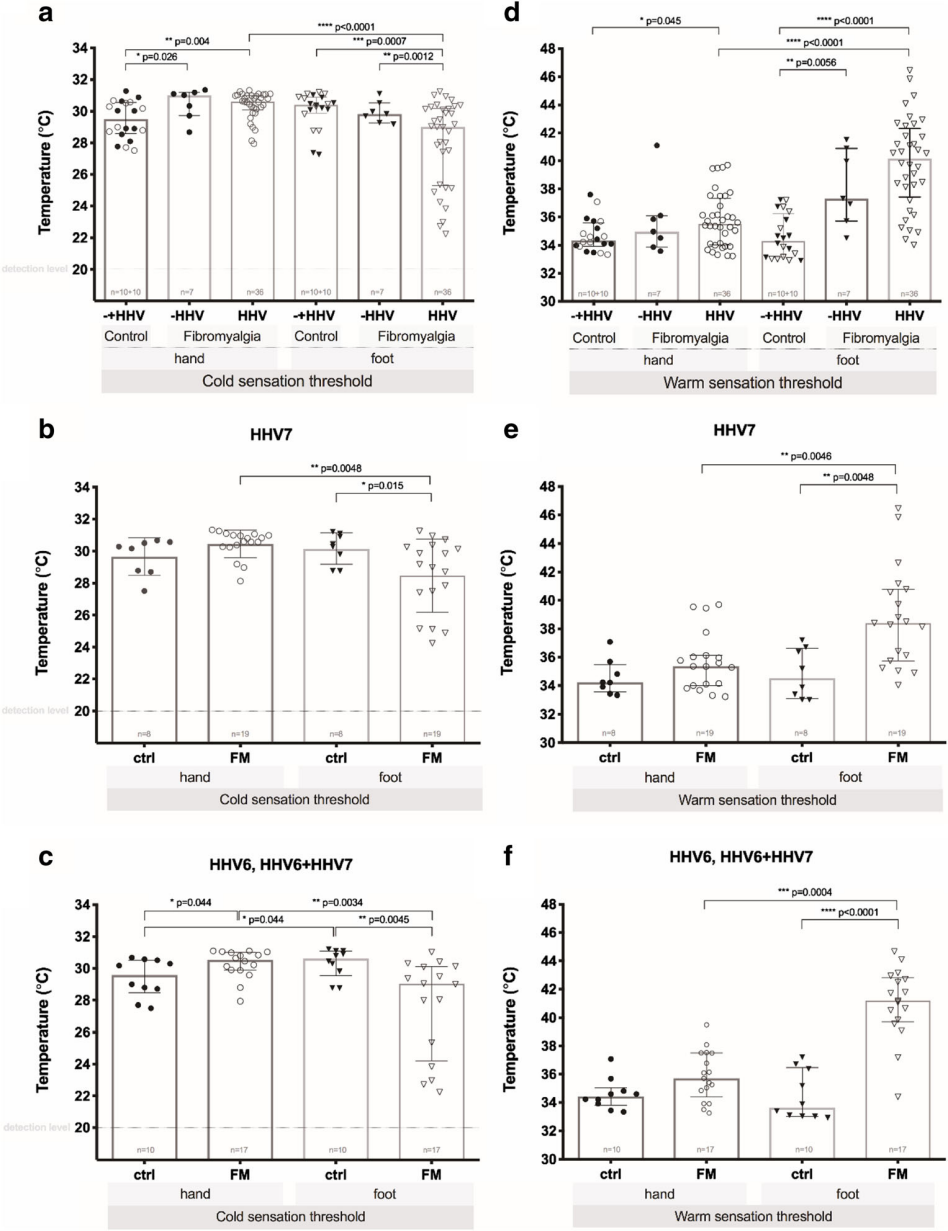
Influence of HHV viral infection on cold (**a**, **b**, **c**) and warm (**d**, **e**, **f**) sensation thresholds on hands and feet in healthy subjects (control, ctrl) and FM patients. − HHV, absent of viral infection; HHV, viral infection (type 6, type 7, or dual 6 + 7). Control group (**a**) is represented by both − HHV (filled symbol) and HHV (empty symbol) subjects. Results are displayed as median and interquartile range (IQR) as dispersion. Fine dotted line shows detection level

**Fig. 5:**
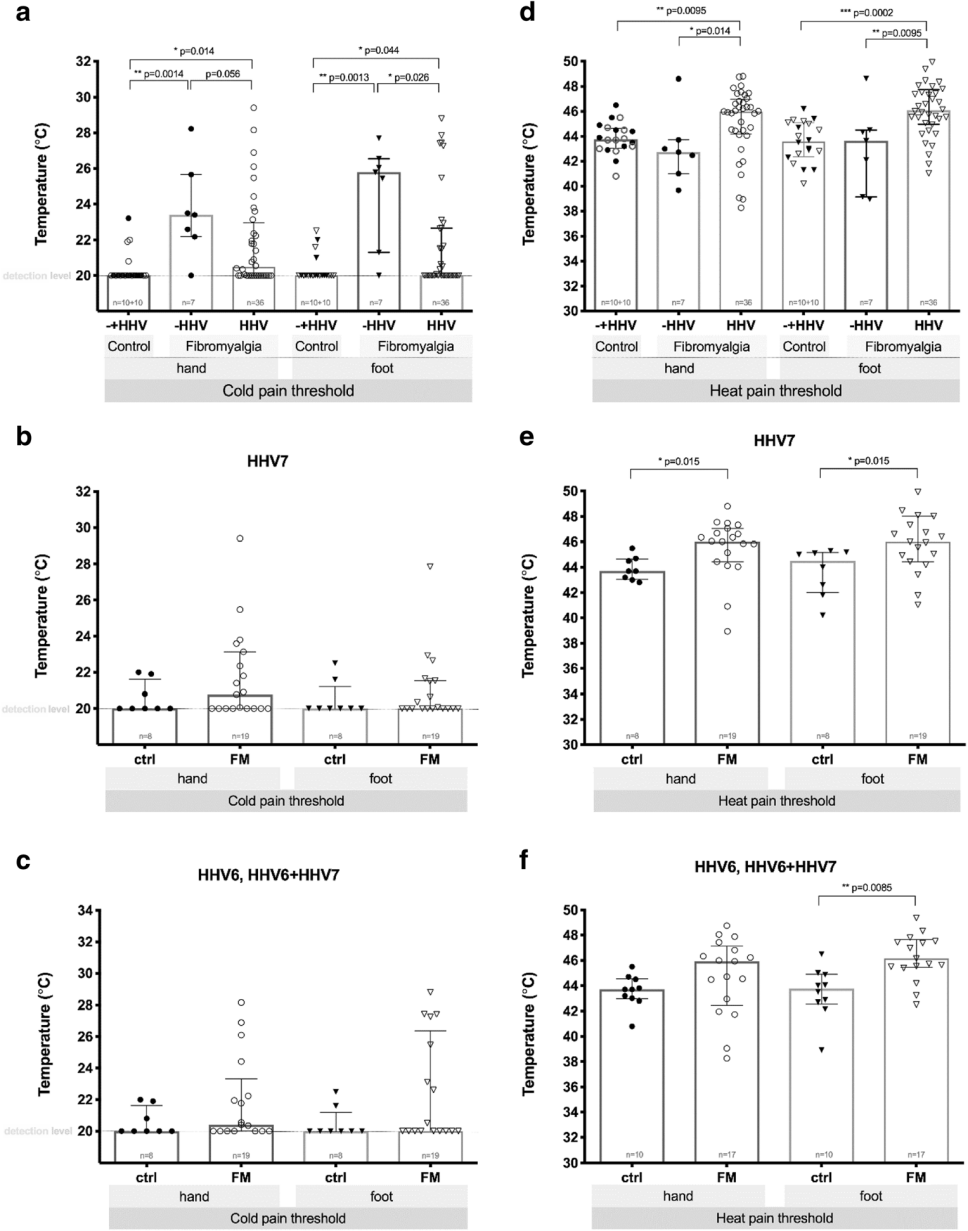
Influence of HHV viral infection on cold (**a**, **b**, **c**) and heat (**d**, **e**, **f**) pain thresholds on hands and feet in healthy subjects (control, ctrl) and FM patients. − HHV, absent of viral infection; HHV, viral infection (type 6, type 7, or dual 6 + 7). Control group (**a**) is represented by both − HHV (filled symbol) and HHV (empty symbol) subjects. Results are displayed as median and interquartile range (IQR) as dispersion. Fine dotted line shows detection level

**Fig. 6:**
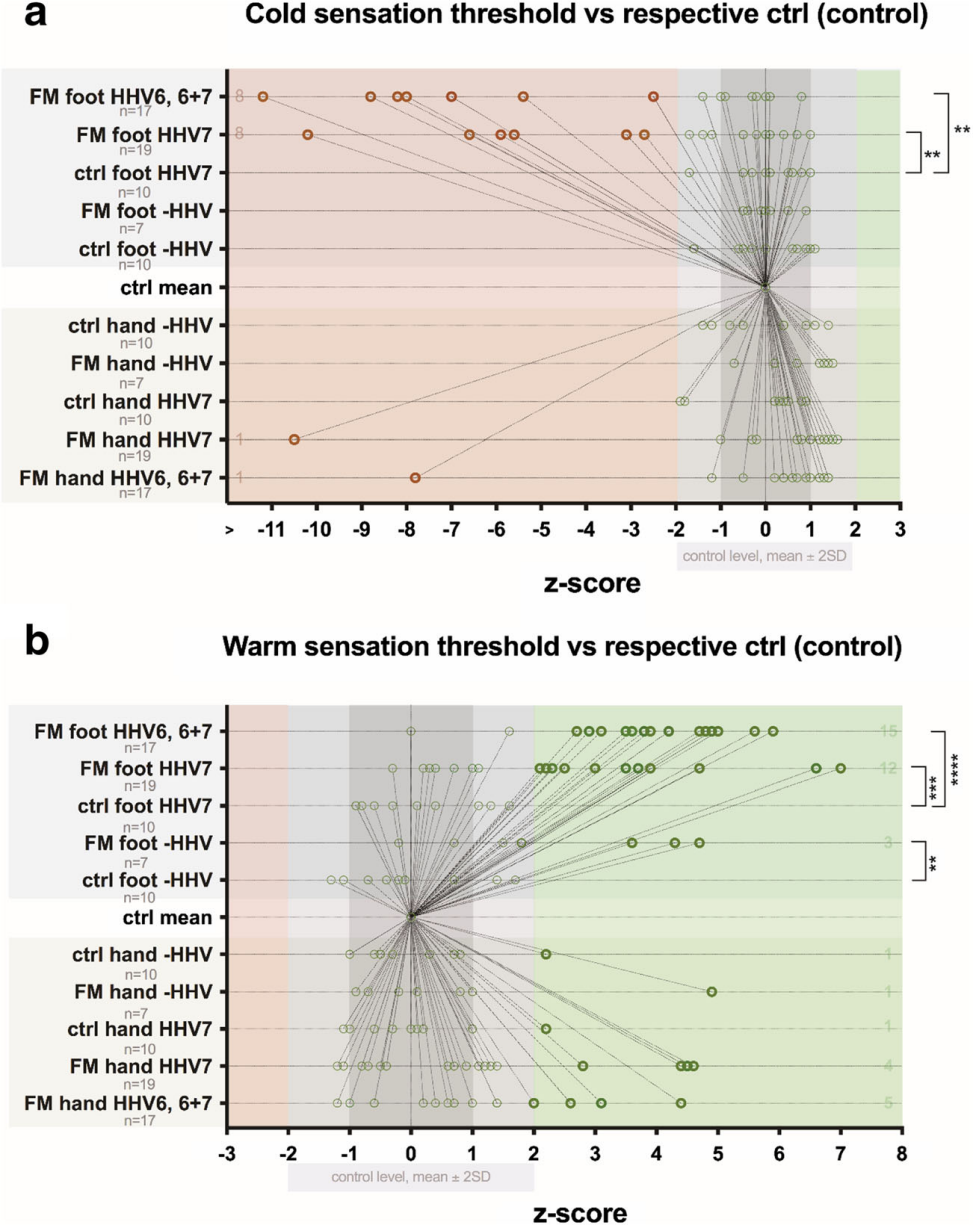
Influence of HHV viral infection on distribution of *z*-scores in healthy subjects (control) and FM patients. **a** Cold sensation threshold. **b** Warm sensation threshold. *Z*-values that significantly differ are marked as bold shape

**Fig. 7:**
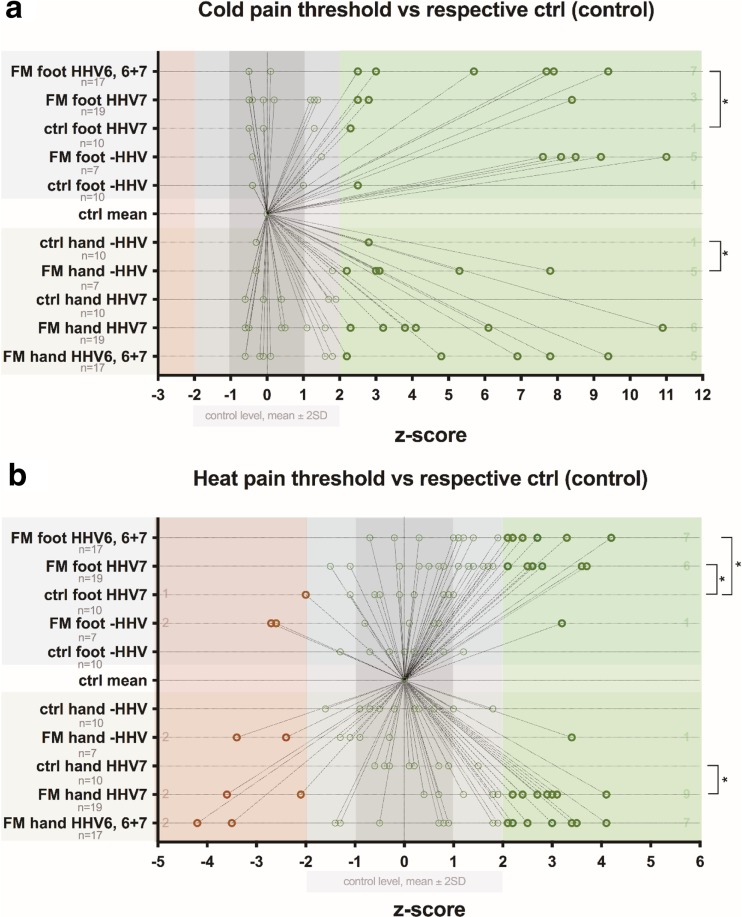
Influence of HHV viral infection on distribution of *z*-scores in healthy subjects (control) and FM patients. **a** Cold pain threshold. **b** Heat pain threshold. *Z*-values that significantly differ are marked as bold shape

A statistically significant (*p* < 0.01) moderate positive correlation (*r* = 0.410) was found between A delta and C nerve fiber damage severity and HHV-6 infection, QST changes were more significant in patients who had persistent HHV-6 infection in active or latent phase. We did not find any correlation between A delta and C nerve fiber damage and the presence of HHV-7 infection markers. There was also no correlation between the presence of viral infection markers and symptom severity according to the FIQ and 2010 ACR preliminary diagnostic criteria for FM. The influence of HHV viral infection on cold and warm sensation and pain threshold is visually shown in Figs. 4 and 5. The influence of HHV viral infection on the distribution of *z*-scores in the control group and FM patients can be seen in Figs. 6 and 7.

**Fig. 8:**
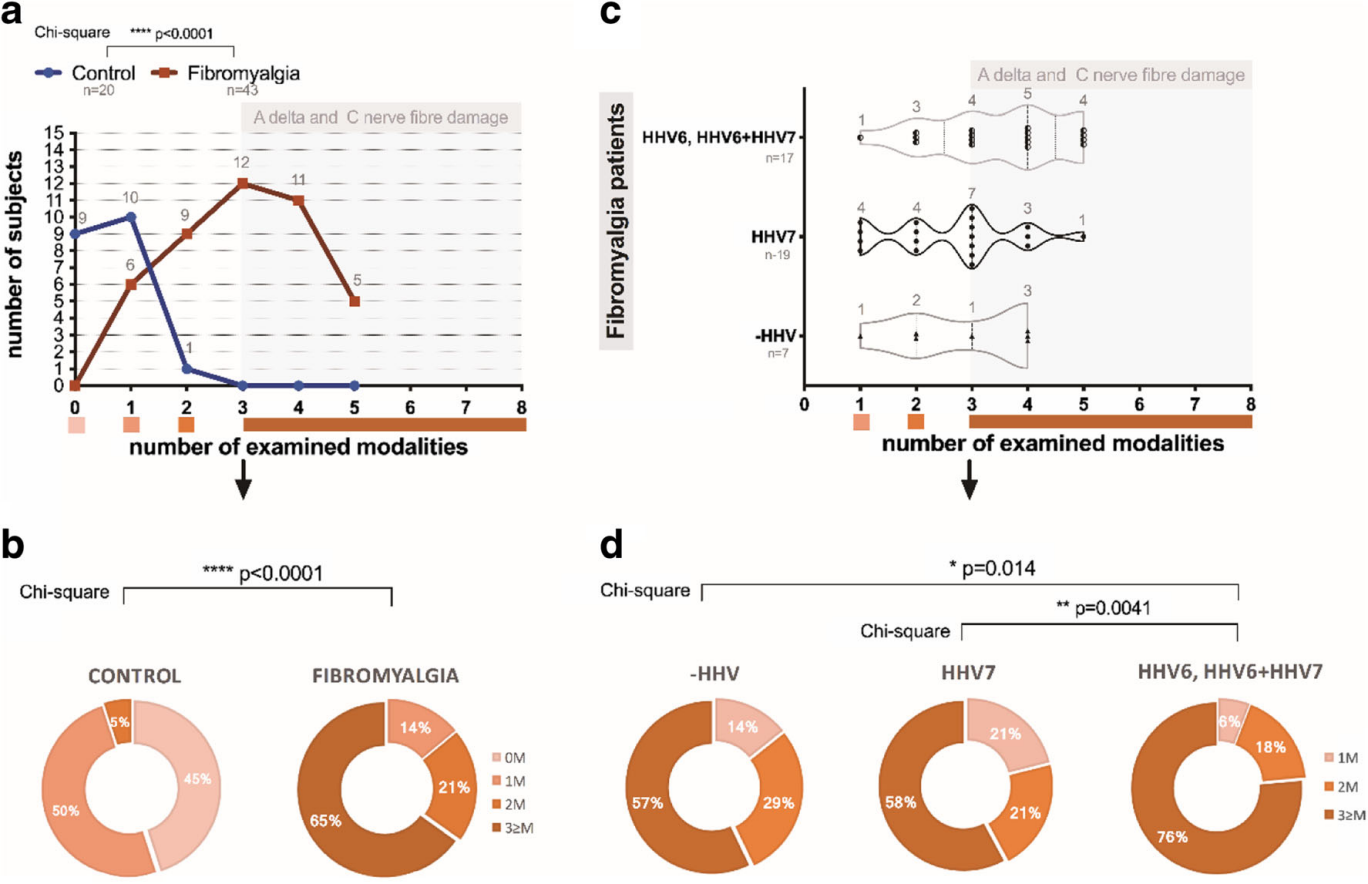
Distribution of examined and changed modalities (out of 8) with respect to FM and to HHV6 and HHV7 infection. **a** Manifestation of modalities in healthy control subjects and FM patients. **b** Distribution of modalities across FM patients. **c** Importance of HHV6 and HHV7 infection and HHV6-HHV7 co-infection on development of sensory dysfunction in FM patients. **d** Distribution of modalities across FM patients regarding HHV6 and HHV7 infection and HHV6-HHV7 co-infection

## Discussion

In this study, clinical characteristics of FM patients, manifested by diffuse musculoskeletal pain and additional somatic symptoms like fatigue, sleepiness, emotional disturbance, depression, cognitive symptoms, gastrointestinal symptoms, and headache, were described. Although inflammatory, autoimmune disorders, and infections have been mentioned as etiological factors in FM development, there are very little data supporting such hypothesis (Borchers and Gershwin 2015). We did not review the mechanism of action of nerve damage in patients with FM and HHV infections because our main focus was on the phenotypic aspect of the disease.

To evaluate peripheral nerve damage in FM patients with HSV-6 and 7, quantitative sensory testing was performed. The QST results of FM patients in all modalities are shown to be more variable than in the control group. We analyzed the average thermal QST results in feet and arms separately because most small fiber neuropathies occur in a length-dependent fashion and primarily affect the lower extremities (Hovaguimian and Gibbons 2011; Themistocleus et al. 2014). FM patients demonstrated significant differences in thermal QST parameters in comparison with healthy controls.

Many previous studies have shown significant differences in thermal QST compared with a control group, but the results differ. Some studies report increased thermal sensitivity in FM patients while others find decreased thermal sensitivity in FM patients compared with that in the control group (Klauenberg et al. 2008; Pavlakovic and Petzke 2010; Plauf et al. 2009). For warm perception, C fibers are responsible for deep and burning pain, and A delta fibers for cold sensation and sharp pain, which indicates light A delta and C nerve fiber damage.

No data exist on the role of HHV-6 and HHV-7 infection in the development of FM, although HHV-6 and HHV-7 are ubiquitous viruses with immunomodulating properties and are potential pathogens to the nervous system (Lusso 2006). In our research, we found nerve fiber damage because of HHV-6; however, no correlation between A delta and C nerve fiber damage and HHV-7 infection was found. An exact mechanism on how HHV-6 virus influenced the nervous system and why there was no correlation between HHV-7 and nerve fiber damage was not analyzed.

The results of our study show no difference in the detection frequency of persistent infection between FM patients and control group individuals; however, higher HHV-6 and HHV-7 load, as well as infection in active phase, were detected only in patients with FM. Statistically significant (*p* < 0.01) moderate positive correlation (*r* = 0.410) was observed between quantitation of changes in QST thermal modalities and HHV-6 infection; QST changes were more significant in patients who have persistent HHV-6 infection in an active or latent phase.

In this study, we found that FM patients have a high level of fatigue (based on the Fatigue Severity Scale and 2010 ACR diagnostic criteria for FM). This finding is consistent with previous studies indicating that between 20 and 70% of FM patients also have chronic fatigue syndrome according to the diagnostic criteria and between 35 and 70% patients with chronic fatigue syndrome correspond to FM diagnostic criteria (Aaron et al. 2000).

## Conclusions

There was a higher detection frequency of persistent HHV-6 and HHV-7 infection in FM patients than in control group individuals. A higher HHV-6 and HHV-7 load and infection in active phase was detected only in patients with FM. Statistically significant correlation was observed between quantitation of changes in QST thermal modalities and HHV-6 infection. There was no correlation between A delta and C nerve fiber damage and HHV-7 infection.
